# Association between metabolic dysfunction-associated fatty liver disease and risk of incidence of gallstones, evidence from a 5-year railway safety staff retrospective cohort study

**DOI:** 10.3389/fmed.2025.1687078

**Published:** 2025-12-09

**Authors:** Liqing Wei, Huihao Wang, Ying Li, Jie Cai, Wen Shang, Hao Liu

**Affiliations:** 1 Department of Blood Transfusion, Wuhan Wuchang Hospital, Wuhan University of Science and Technology, Wuhan, China; 2 Wuhan Children's Hospital, Wuhan Maternal and Child Healthcare Hospital, Tongji Medical College, Huazhong University of Science and Technology, Wuhan, China; 3 Physical Examination Center, Renmin Hospital of Wuhan University, Wuhan, China; 4 Physical Examination Center, Wuhan Wuchang Hospital, Wuhan University of Science and Technology, Wuhan, China; 5 Department of Orthopedics, Wuhan Wuchang Hospital, Wuhan University of Science and Technology, Wuhan, China

**Keywords:** fatty liver, gallstones, cohort, railway safety staff, survival

## Abstract

**Background:**

Current evidence from cohort studies on the association between metabolic dysfunction-associated fatty liver disease (MAFLD) and gallstone formation remains limited. This 5-year retrospective cohort study aimed to evaluate the impact of MAFLD on gallstone incidence among railway safety staff.

**Methods:**

Data were obtained from the physical examination data of railway safety staff at Wuchang Hospital, Wuhan City, Hubei Province, China. The diagnosis of MAFLD was consistent with the international expert consensus. Independent associations between MAFLD and participants' risk of gallstone incidence were assessed by multiple Cox regression. Stratified analyses and interaction tests were performed to investigate whether covariates modified the association between MAFLD and gallstone incidence.

**Results:**

Among 1,422 participants, the risk of gallstone incidence was 9.27% (37/399) higher in the exposed group with MAFLD than in the control group 5.67% (58/1,023). Multiple cox regression showed that people with MAFLD were associated with a risk of gallstone incidence (HR = 1.71, 95% CI = 1.13–2.58). Stratified analysis by sex showed that men with MAFLD had a significantly increased risk (HR = 1.54, 95% CI: 1.00–2.38, *P* = 0.050), whereas women did not reach statistical significance (*P* = 0.605), but the sex interaction was not significant (*P*-interaction = 0.664). In age-stratified analyses, both the 45–60 years group (HR = 1.58, *P* = 0.078) and the 30–45 years group (HR = 1.89, *P* = 0.179) showed a trend toward increased risk, but the interaction was not statistically different (*P*-interaction = 0.270).

**Conclusions:**

There is a significant association between MAFLD and the incidence of gallstones. Attention should be paid to the prevention and treatment of MAFLD to promote the health of railway safety staff and reduce the burden ofanalysis gallstone-related diseases.

## Background

Gallstone disease (GD) has become a major global health issue, affecting 10%−15% of adults in Western countries, with a rapidly rising incidence in Asia linked to changes in lifestyle and diet (1–3) As one of the most common gastrointestinal disorders, GD imposes a significant economic burden, especially when complications necessitate surgical interventions such as cholecystectomy (4, 5). Although mortality of gallstone is relatively low, its high morbidity and associated costs place substantial pressure on families and healthcare systems, especially in populous developing countries like China (6, 7). In China, the epidemiology of liver disease has shifted markedly, while viral hepatitis was historically the leading cause of liver-related morbidity, vaccination and antiviral advances have reduced its burden. Meanwhile, metabolic dysfunction-associated fatty liver disease (MAFLD) has become the most prevalent chronic liver condition, affecting over 29.2% of the population (8–10). MAFLD indicates that this disease is closely related to metabolic dysfunction and metabolic syndrome, thereby better reflecting the etiology of fatty liver and shifting from exclusion diagnosis to diagnosis based on positive criteria (11).

The biological association between MAFLD and GD is reasonable, as they share a common metabolic disorder mechanism. Both conditions are strongly associated with obesity, insulin resistance, type 2 diabetes, and dyslipidemia, which are key phenotypic components of metabolic syndrome (12–14). From a mechanistic standpoint, MAFLD may promote gallstone formation through several pathways: hepatic insulin resistance activates sterol regulatory element-binding protein-2 (SREBP-2), enhancing cholesterol synthesis and leading to cholesterol-supersaturated bile (15, 16). Additionally, inflammatory mediators derived from visceral adipose tissue in MAFLD patients can impair gallbladder motility by downregulating contractile protein expression and reducing acetylcholine sensitivity in smooth muscle, thereby predisposing to stone formation (17). Emerging epidemiological evidence suggests a significant association between MAFLD and an increased risk of GD. A meta-analysis of eight studies involving 326,922 participants confirmed this association. MAFLD patients had a higher incidence of GD (OR: 1.71, CI: 1.63–1.79, *P* < 0.001) and this risk may be increased in females (OR: 4.18; CI: 1.21–14.37) and those with high BMI (OR: 1.80; CI: 1.36–2.56) (18), the results of a cohort study showed that GD and cholecystectomy were independently associated with MAFLD (OR = 1.75, 95% CI: 1.43–2.15 for GD; OR = 2.77, 95% CI: 2.01–3.83 compared with no GD) (19). At the same time, studies have found a bidirectional association between MAFLD and incidence of gallstones. MAFLD and its severity are independently associated with an increased incidence of gallstones, while gallstones and cholecystectomy are also associated with the incidence of MAFLD (20, 21). These bidirectional associations suggest a complex interplay that may involve shared metabolic, inflammatory, and possibly gut-liver axis mechanisms.

Despite growing evidence linking MAFLD with GD, significant knowledge gaps persist. Current evidence, predominantly from cross-sectional studies, limits causal inference regarding the relationship. Furthermore, the generalizability of findings across diverse populations is constrained by genetic, dietary, and lifestyle variations, and the potential confounding by metabolic comorbidities remains a challenge. Railway safety staff present an ideal model for investigation. They frequently work in shifts, which are factors contributing to metabolic abnormalities associated with MAFLD and gallstones. Additionally, Railway safety staff is exposed to distinct occupational stressors that may modulate liver metabolism via neuroendocrine pathways. Crucially, the comprehensive and standardized annual health examination system within the railway sector offers robust, longitudinal data, effectively minimizing socioeconomic confounders due to the population's homogeneity in work models and healthcare access. The 5-year follow-up period is sufficient to observe the complete pathological process of metabolic abnormalities to gallstone formation. This study aims to investigate the association between MAFLD and GD based on the data of retrospective railway safety staff for up to 5 years, providing a scientific basis for the prevention of GD.

## Methods

### Source of data

**Figure 1:**
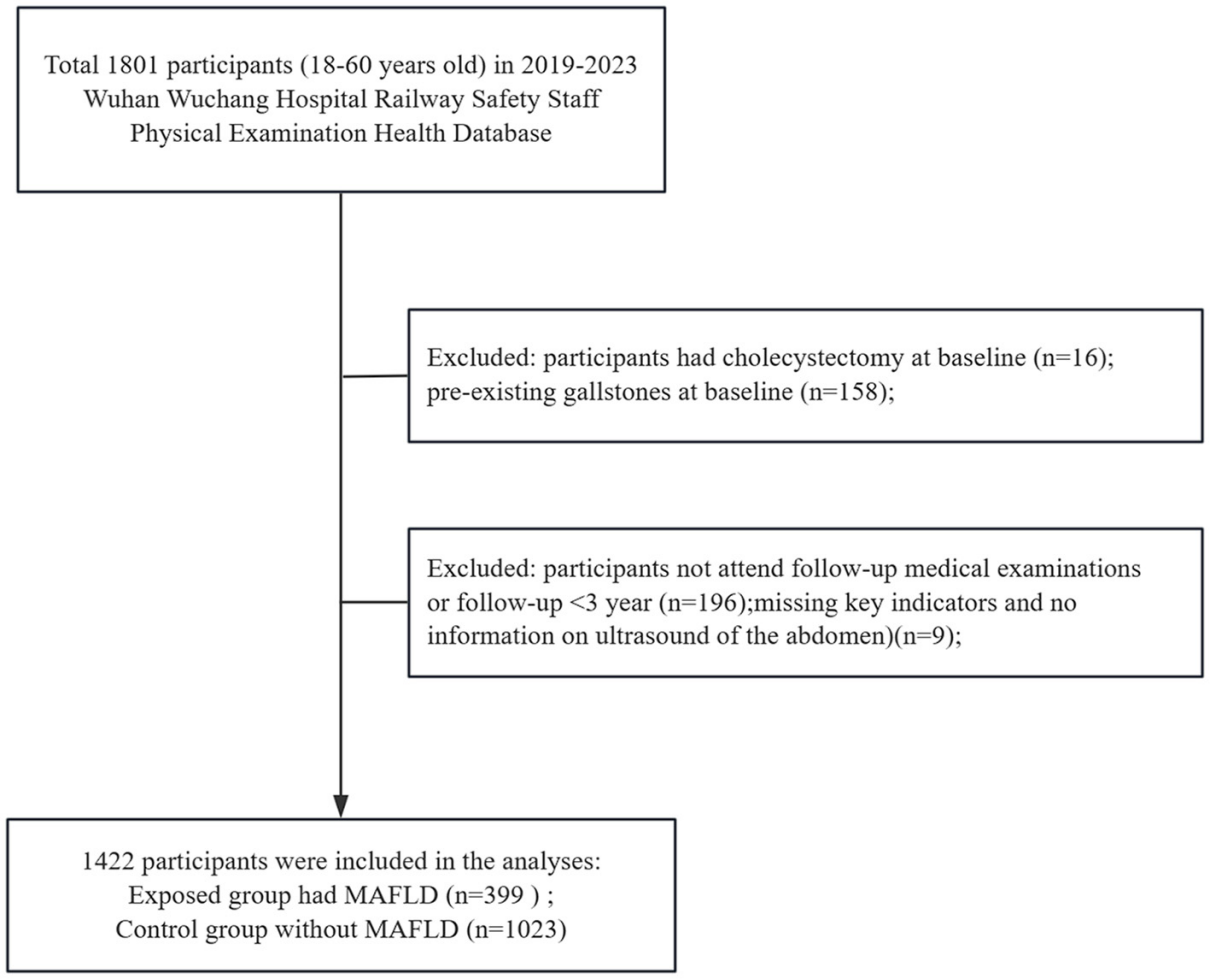
Flowchart of the study population.

The retrospective cohort study using de-identified annual physical-examination records of railway safety staff from Wuchang Hospital, Wuhan, China, covering 85% of the railway safety staff in Wuhan prefecture and its three satellite cities between 1 January 2019 and 31 December 2023. This database records detailed information on population statistics, laboratory tests, imaging data, vital signs, and disease diagnosis. Due to the lack of protected information in the database and the anonymity of patients, a waiver of informed consent was granted (Figure 1).

### Identification of MAFLD

According to the criteria in the latest international diagnostic consensus (10) diagnostic criteria for MAFLD in this study were based on imaging and blood biomarker evidence of hepatic fat accumulation (hepatocellular steatosis) in combination with one of three conditions: overweight/obesity, type 2 diabetes mellitus, and metabolic dysfunction, in accordance with the criteria set forth in the most recent international diagnostic consensus. Metabolic dysfunction is defined as the presence of at least two risk factors for metabolic abnormalities. These factors include: waist circumference: ≥90 and 80 cm in Asian men and women, respectively; blood pressure: ≥130/85 mmHg or on blood pressure-lowering medication; blood triglycerides: ≥1.7 mmol/L or on lipid-lowering medication; plasma high-density lipoprotein (Hdl) cholesterol: < 1.0 and 1.3 mmol/L in men and women, respectively, or on lipid-lowering medication; prediabetes fasting glucose: 5.6–6.9 mmol/L or 2 h postprandial glucose 7.8–11.0 mmol/L or glycated hemoglobin 5.7%−6.4%; homeostasis model assessment of insulin resistance index: ≥2.5; high-sensitivity C-reactive protein: >2 mg/L.

### Assessment of covariates

Clinical covariates potentially associated with MAFLD and gallstones were retrieved from 2019–2023 Wuhan Wuchang Hospital Railway Safety Staff Physical Examination Health Database. Demographic variables for each patient included age and sex. Body mass index (BMI) was calculated as weight (kg)/height^2^ (m^2^), and the presence of comorbidities was defined according to pre-index date ICD-10 codes, defined using dichotomous variables, including diabetes mellitus, hypertension. Laboratory results included lipid indices: TC (total cholesterol), TG (triglycerides), LDL-C (low-density lipoprotein cholesterol), HDL-C (high-density lipoprotein cholesterol), which were converted into categorical indices according to the Chinese lipid management guidelines in order to make the lipid indices more clinically informative (22); fasting blood glucose; renal function indices of creatinine and uric acid; liver function indices of Alanine aminotransferase (ALT), Aspartate Aminotransferase (AST), Direct Bilirubin (DBil), Total Bilirubin (TBil). To improve the clinical significance of the results and to detect potential non-linear relationships, continuous variables were transformed into categorical variables based on their distribution within the cohort. Specifically, they were divided into four quartiles (Q1–Q4), where Q1 represents the lowest 25% of values and Q4 the highest 25%.

### Statistical analysis

The baseline characteristics of the research subjects are represented as the mean ± standard deviation of continuous variables, while categorical variables are represented as numbers (percentages). Compare the values of each variable between two groups using independent *t*-test for continuous variables and chi square test for categorical variables. We used Cox proportional hazards regression analysis to evaluate the incidence of new cases of MAFLD and gallstones, as well as the differences in this association between different subgroups. The results are expressed as hazard ratio (HR) [95% confidence interval (CI)]. Compare the HR (95% CI) of gallstones in the MAFLD group with that in the non fatty liver group as a reference. Two-sided *P* < 0.05 was considered statistically significant. Perform statistical analysis using R 4.5.1 software.

## Results

This study enrolled a total of 1,422 participants (Table 1). The 399 participants (28.06%) diagnosed with MAFLD at baseline were classified as the Exposed group, with the remaining participants forming the Control group (*n* = 1,023, 71.94%). Median follow-up was 4.12 years, yielding 4 750 person-years. Compared with controls, the MAFLD group differed significantly (*P* < 0.05) in sex, age, BMI, hypertension, diabetes, TC, LDL-C and HDL-C categories, creatinine, uric acid, TG, FBG, ALT, AST and DBil, whereas total bilirubin levels were comparable (*P* > 0.05).

**Table 1:** Baseline characteristics of participants.

**Variables**	**Total (*n* = 1,422)**	**Control (*n* = 1,023)**	**Exposed (*n* = 399)**	**Statistic**	***P*-value**
**Sex**, ***n*** **(%)**					
Male	1,168 (82.14)	780 (76.25)	388 (97.24)	χ^2^ = 86.25	**< 0.001**
Female	254 (17.86)	243 (23.75)	11 (2.76)		
**Age**, ***n*** **(%)**					
18–30	391 (27.50)	318 (31.09)	73 (18.30)	χ^2^ = 31.93	**< 0.001**
30–45	477 (33.54)	347 (33.92)	130 (32.58)		
45–60	554 (38.96)	358 (35.00)	196 (49.12)		
**BMI**, ***n*** **(%)**					
Normal	607 (42.69)	572 (55.91)	35 (8.77)	χ^2^ = 389.64	**< 0.001**
Underweight	46 (3.23)	46 (4.50)	0 (0.00)		
Overweight	574 (40.37)	351 (34.31)	223 (55.89)		
Obese	195 (13.71)	54 (5.28)	141 (35.34)		
**Diabetes**, ***n*** **(%)**					
No	1,107 (77.85)	877 (85.73)	230 (57.64)	χ^2^ = 131.28	**< 0.001**
Yes	315 (22.15)	146 (14.27)	169 (42.36)		
**Hypertension**, ***n*** **(%)**					
No	1,141 (80.24)	884 (86.41)	257 (64.41)	χ^2^ = 87.63	**< 0.001**
Yes	281 (19.76)	139 (13.59)	142 (35.59)		
**TC**, ***n*** **(%)**					
Normal	1,027 (72.22)	792 (77.42)	235 (58.90)	χ^2^ = 52.61	**< 0.001**
Borderline high	290 (20.39)	177 (17.30)	113 (28.32)		
High	105 (7.38)	54 (5.28)	51 (12.78)		
**LDL**, ***n*** **(%)**					
Ideal	647 (45.50)	515 (50.34)	132 (33.08)	χ^2^ = 57.84	**< 0.001**
Optimal	495 (34.81)	353 (34.51)	142 (35.59)		
Borderline high	206 (14.49)	117 (11.44)	89 (22.31)		
High	74 (5.20)	38 (3.71)	36 (9.02)		
**HDL**, ***n*** **(%)**					
Normal	1,240 (87.20)	930 (90.91)	310 (77.69)	χ^2^ = 44.91	**< 0.001**
Abnormal	182 (12.80)	93 (9.09)	89 (22.31)		
**Creatinine. quantile**, ***n*** **(%)**					
Mean ± SD (μmol/L)	72.87 ± 13.46	72.30 ± 14.32	74.33 ± 10.83	*t* = −2.89	**0.004**
Q1	355 (24.96)	288 (28.15)	67 (16.79)	χ^2^ = 23.56	**< 0.001**
Q2	347 (24.40)	227 (22.19)	120 (30.08)		
Q3	363 (25.53)	251 (24.54)	112 (28.07)		
Q4	357 (25.11)	257 (25.12)	100 (25.06)		
**Uric acid. quantile**, ***n*** **(%)**					
Mean ± SD (μmol/L)	387.50 ± 98.97	368.63 ± 95.85	435.90 ± 90.10	*t* = −12.09	**< 0.001**
Q1	356 (25.04)	324 (31.67)	32 (8.02)	χ^2^ = 130.06	**< 0.001**
Q2	355 (24.96)	268 (26.20)	87 (21.80)		
Q3	355 (24.96)	241 (23.56)	114 (28.57)		
Q4	356 (25.04)	190 (18.57)	166 (41.60)		
**TG. quantile**, ***n*** **(%)**					
Mean ± SD (μmol/L)	1.83 ± 1.67	1.49 ± 1.24	2.70 ± 2.23	*t* = −10.27	**< 0.001**
Q1	351 (24.68)	340 (33.24)	11 (2.76)	χ^2^ = 276.74	**< 0.001**
Q2	359 (25.25)	297 (29.03)	62 (15.54)		
Q3	355 (24.96)	229 (22.39)	126 (31.58)		
Q4	357 (25.11)	157 (15.35)	200 (50.13)		
**FBG. quantile**, ***n*** **(%)**					
Mean ± SD (mmol/L)	5.21 ± 1.48	5.04 ± 1.27	5.64 ± 1.83	*t* = −6.01	**< 0.001**
Q1	356 (25.04)	279 (27.27)	77 (19.30)	χ^2^ = 81.30	**< 0.001**
Q2	351 (24.68)	282 (27.57)	69 (17.29)		
Q3	354 (24.89)	268 (26.20)	86 (21.55)		
Q4	361 (25.39)	194 (18.96)	167 (41.85)		
**ALT. quantile**, ***n*** **(%)**					
Mean ± SD (μmol/L)	30.56 ± 25.28	24.72 ± 18.94	45.52 ± 32.37	*t* = −12.06	**< 0.001**
Q1	351 (24.68)	337 (32.94)	14 (3.51)	χ^2^ = 289.68	**< 0.001**
Q2	358 (25.18)	300 (29.33)	58 (14.54)		
Q3	356 (25.04)	236 (23.07)	120 (30.08)		
Q4	357 (25.11)	150 (14.66)	207 (51.88)		
**AST. quantile**, ***n*** **(%)**					
Mean ± SD (μmol/L)	25.46 ± 12.83	23.15 ± 8.93	31.38 ± 18.28	*t* = −8.60	**< 0.001**
Q1	355 (24.96)	320 (31.28)	35 (8.77)	χ^2^ = 146.95	**< 0.001**
Q2	347 (24.40)	261 (25.51)	86 (21.55)		
Q3	361 (25.39)	263 (25.71)	98 (24.56)		
Q4	359 (25.25)	179 (17.50)	180 (45.11)		
**DBil. quantile**, ***n*** **(%)**					
Mean ± SD (μmol/L)	3.00 ± 1.19	3.08 ± 1.24	2.77 ± 1.02	*t* = 4.87	**< 0.001**
Q1	339 (23.84)	225 (21.99)	114 (28.57)	χ^2^ = 17.09	**< 0.001**
Q2	331 (23.28)	229 (22.39)	102 (25.56)		
Q3	360 (25.32)	258 (25.22)	102 (25.56)		
Q4	392 (27.57)	311 (30.40)	81 (20.30)		
**TBil. quantile**, ***n*** **(%)**					
Mean ± SD (μmol/L)	15.28 ± 6.23	15.41 ± 6.34	14.94 ± 5.93	*t* = 1.26	0.206
Q1	350 (24.61)	249 (24.34)	101 (25.31)	χ^2^ = 1.27	0.737
Q2	350 (24.61)	246 (24.05)	104 (26.07)		
Q3	359 (25.25)	260 (25.42)	99 (24.81)		
Q4	363 (25.53)	268 (26.20)	95 (23.81)		

t, t-test, χ^2^, chi-square test; SD, standard deviation. Bold: Statistically different.

Multivariable cox regression (Table 2) showed MAFLD remained independently associated with incident gallstones after adjustment for sex, age, BMI and metabolic variables (HR 1.71; 95 % CI 1.13–2.58; *P* = 0.011). Age-stratified analysis showed that those aged 45–60 years had a significantly higher risk than the reference group aged 18–30 years (HR=2.82, 95% CI: 1.64–4.84, *P* < 0.001). Among metabolic related factors, overweight (HR = 1.77, 95% CI: 1.12–2.79, *P* = 0.014), diabetes (HR = 1.97, 95% CI: 1.29–3.00, *P* = 0.002), and hypertension (HR = 2.60, 95% CI: 1.71–3.97, *P* < 0.001) showed independent predictive effects. Males showed a trend of increased risk compared to females (HR = 1.83,95% CI: 0.97–3.42, *P* = 0.060), but did not reach statistical significance. Among the lipid indices, total cholesterol (TC) and low-density lipoprotein (LDL) stratification did not show a significant association, while the highest quartile group (Q4) of fasting glucose had a 93% increased risk (HR = 1.93, 95% CI: 1.08–3.42, *P* = 0.025). No significant associations were observed across quartiles of ALT, AST or bilirubin (all *P* > 0.05).

**Table 2:** Risk of incidence of gallstones according to the MAFLD status.

**Variables**	**β**	**S.E**	***Z***	***P*-value**	**HR (95% CI)**
**MAFLD**					
No					1.00 (Reference)
Yes	0.53	0.21	2.54	0.011	1.71 (1.13–2.58)
**Sex**					
Female					1.00 (Reference)
Male	0.60	0.32	1.88	0.060	1.83 (0.97–3.42)
**Age**					
18–30					1.00 (Reference)
30–45	−0.13	0.33	−0.40	0.688	0.87 (0.45–1.68)
45–60	1.04	0.28	3.76	< 0.001	2.82 (1.64–4.84)
**BMI**					
Normal					1.00 (Reference)
Underweight	−0.89	1.02	−0.87	0.382	0.41 (0.06–3.02)
Overweight	0.57	0.23	2.45	0.014	1.77 (1.12–2.79)
Obese	0.48	0.31	1.54	0.124	1.61 (0.88–2.95)
**Diabetes**					
No					1.00 (Reference)
Yes	0.68	0.22	3.13	0.002	1.97 (1.29–3.00)
**Hypertension**					
No					1.00 (Reference)
Yes	0.96	0.21	4.45	< 0.001	2.60 (1.71–3.97)
**TC**					
Normal					1.00 (Reference)
Borderline high	−0.06	0.27	−0.24	0.812	0.94 (0.55–1.60)
High	0.45	0.34	1.32	0.188	1.56 (0.80–3.03)
**LDL**					
Ideal					1.00 (Reference)
Optimal	0.22	0.23	0.95	0.342	1.24 (0.80–1.93)
Borderline high	0.09	0.32	0.27	0.785	1.09 (0.58–2.05)
High	−0.01	0.53	−0.03	0.977	0.99 (0.35–2.76)
**HDL**					
Normal					1.00 (Reference)
Abnormal	0.39	0.35	1.10	0.269	1.97 (1.29–3.00)
**Creatinine, quantile**					
Q1					1.00 (Reference)
Q2	−0.14	0.30	−0.45	0.649	0.87 (0.48–1.58)
Q3	−0.03	0.29	−0.09	0.930	0.97 (0.55–1.73)
Q4	0.23	0.28	0.83	0.405	1.26 (0.73–2.17)
**Uric acid, quantile**					
Q1					1.00 (Reference)
Q2	0.01	0.30	0.04	0.967	1.01 (0.56–1.82)
Q3	0.12	0.29	0.42	0.673	1.13 (0.64–1.98)
Q4	0.08	0.29	0.26	0.791	1.08 (0.61–1.92)
**TG, quantile**					
Q1					1.00 (Reference)
Q2	0.34	0.30	1.15	0.250	1.41 (0.79–2.52)
Q3	0.27	0.30	0.90	0.366	1.31 (0.73–2.37)
Q4	0.14	0.31	0.46	0.649	1.15 (0.62–2.13)
**FBG, quantile**					
Q1					1.00 (Reference)
Q2	−0.02	0.33	−0.06	0.955	0.98 (0.52–1.86)
Q3	0.51	0.29	1.75	0.079	1.67 (0.94–2.94)
Q4	0.66	0.29	2.23	0.025	1.93 (1.08–3.42)
**ALT, quantile**					
Q1					1.00 (Reference)
Q2	0.16	0.29	0.57	0.569	1.18 (0.67–2.07)
Q3	0.22	0.28	0.77	0.439	1.24 (0.72–2.17)
Q4	−0.29	0.32	−0.89	0.375	0.75 (0.40–1.41)
**AST, quantile**					
Q1					1.00 (Reference)
Q2	0.21	0.28	0.75	0.454	1.23 (0.72–2.11)
Q3	−0.32	0.31	−1.02	0.306	0.73 (0.39–1.34)
Q4	0.04	0.29	0.13	0.899	1.04 (0.59–1.83)
**DBil, quantile**					
Q1					1.00 (Reference)
Q2	−0.10	0.28	−0.35	0.727	0.91 (0.52–1.57)
Q3	−0.27	0.29	−0.94	0.347	0.76 (0.43–1.35)
Q4	−0.27	0.28	−0.94	0.346	0.77 (0.44–1.33)
**TBil, quantile**					
Q1					1.00 (Reference)
Q2	0.32	0.31	1.02	0.309	1.37 (0.75–2.53)
Q3	0.28	0.31	0.92	0.357	1.33 (0.73–2.44)
Q4	0.41	0.30	1.37	0.171	1.51 (0.84–2.73)

Subgroup analyses showed that the association between MAFLD and risk of incidence of gallstones was heterogeneous across populations (Table 3). Sex stratification showed a significantly increased risk in men with MAFLD (HR = 1.54, 95% CI: 1.00–2.38, *P* = 0.050), while women did not reach statistical significance (*P* = 0.605), but the sex interaction was not significant (*P*-interaction = 0.664). In the age stratification, both the 45–60 years group (HR = 1.58, *P* = 0.078) and the 30–45 years group (HR = 1.89, *P* = 0.179) showed a trend of increased risk, but the interaction was not statistically different (*P*-interaction = 0.270).

**Table 3:** Subgroup analysis.

**Variables**	***n* (%)**	**Control (*n* = 1,023)**	**Exposed (*n* = 399)**	**HR (95% CI)**	***P*-value**	***P* for interaction**
All patients	1,422 (100.00)	58/1,023	37/399	1.71 (1.13–2.58)	**0.011**	
**Sex**						
Female	254 (17.86)	10/243	1/11	1.77 (0.20–15.36)	0.605	0.664
Male	1,168 (82.14)	48/780	36/388	1.54 (1.00–2.38)	0.050	
**Age**						
18–30	391 (27.50)	15/318	2/73	0.58 (0.13–2.55)	0.474	0.270
30–45	477 (33.54)	11/347	8/130	1.89 (0.75–4.76)	0.179	
45–60	554 (38.96)	32/358	27/196	1.58 (0.95–2.65)	0.078	
**BMI**						
Normal	607 (42.69)	28/572	2/35	1.48 (0.35–6.21)	0.593	0.450
Underweight	46 (3.23)	1/46	0/0			
Overweight	574 (40.37)	27/351	21/223	1.21 (0.68–2.13)	0.521	
Obese	195 (13.71)	2/54	14/141	2.99 (0.68–13.27)	0.149	
**Diabetes**						
No	1,107 (77.85)	42/877	20/230	1.94 (1.14–3.30)	**0.015**	0.073
Yes	315 (22.15)	16/146	17/169	0.90 (0.45–1.78)	0.764	
**Hypertension**						
No	1,141 (80.24)	44/884	17/257	1.37 (0.78–2.40)	0.272	0.993
Yes	281 (19.76)	14/139	20/142	1.35 (0.68–2.67)	0.390	
**TC**						
Normal	1,027 (72.22)	45/792	23/235	1.85 (1.12–3.05)	**0.017**	0.645
Borderline high	290 (20.39)	8/177	9/113	1.75 (0.68–4.55)	0.247	
High	105 (7.38)	5/54	5/51	0.83 (0.22–3.08)	0.775	
**LDL**						
Ideal	647 (45.50)	28/515	11/132	1.74 (0.86–3.49)	0.121	0.648
Optimal	495 (34.81)	21/353	18/142	2.19 (1.16–4.10)	**0.015**	
Borderline high	206 (14.49)	7/117	6/89	0.89 (0.28–2.80)	0.842	
High	74 (5.20)	2/38	2/36	1.21 (0.17–8.57)	0.851	
**HDL**						
Normal	1,240 (87.20)	54/930	32/310	1.86 (1.20–2.88)	**0.005**	0.708
Abnormal	182 (12.80)	4/93	5/89	1.49 (0.40–5.56)	0.552	
**Creatinine, quantile**						
Q1	355 (24.96)	16/288	8/67	2.35 (1.00–5.50)	**0.049**	0.711
Q2	347 (24.40)	10/227	10/120	1.96 (0.81–4.70)	0.133	
Q3	363 (25.53)	13/251	10/112	1.79 (0.79–4.09)	0.165	
Q4	357 (25.11)	19/257	9/100	1.22 (0.55–2.71)	0.617	
**Uric acid, quantile**						
Q1	356 (25.04)	15/324	8/32	6.50 (2.75–15.34)	**< 0.001**	0.023
Q2	355 (24.96)	15/268	7/87	1.50 (0.61–3.69)	0.373	
Q3	355 (24.96)	16/241	10/114	1.33 (0.61–2.94)	0.474	
Q4	356 (25.04)	12/190	12/166	1.14 (0.51–2.54)	0.749	
**TG, quantile**						
Q1	351 (24.68)	18/340	1/11	2.10 (0.28–15.72)	0.471	0.395
Q2	359 (25.25)	19/297	9/62	2.32 (1.04–5.15)	**0.039**	
Q3	355 (24.96)	11/229	15/126	2.49 (1.14–5.42)	**0.022**	
Q4	357 (25.11)	10/157	12/200	1.02 (0.44–2.36)	0.966	
**FBG, quantile**						
Q1	356 (25.04)	12/279	8/77	2.62 (1.00–6.49)	**0.051**	0.127
Q2	351 (24.68)	15/282	3/69	0.78 (0.23–2.70)	0.696	
Q3	354 (24.89)	16/268	13/86	2.50 (1.20–5.20)	**0.014**	
Q4	361 (25.39)	15/194	13/167	0.97 (0.46–2.05)	0.945	
**ALT, quantile**						
Q1	351 (24.68)	20/337	2/14	4.38 (1.02–18.78)	**0.047**	0.632
Q2	358 (25.18)	18/300	9/58	2.93 (1.31–6.53)	**0.009**	
Q3	356 (25.04)	15/236	14/120	1.78 (0.86–3.70)	0.120	
Q4	357 (25.11)	5/150	12/207	1.75 (0.62–4.98)	0.292	
**AST, quantile**						
Q1	355 (24.96)	22/320	2/35	0.97 (0.23–4.11)	0.963	0.183
Q2	347 (24.40)	18/261	11/86	2.02 (0.95–4.28)	0.066	
Q3	361 (25.39)	7/263	11/98	3.80 (1.46–9.92)	**0.006**	
Q4	359 (25.25)	11/179	13/180	1.18 (0.53–2.64)	0.685	
**DBil, quantile**						
Q1	339 (23.84)	13/225	14/114	2.16 (1.01–4.61)	**0.047**	0.260
Q2	331 (23.28)	12/229	12/102	2.34 (1.05–5.22)	**0.037**	
Q3	360 (25.32)	13/258	8/102	1.53 (0.64–3.70)	0.342	
Q4	392 (27.57)	20/311	3/81	0.66 (0.20–2.22)	0.502	
**TBil. quantile**						
Q1	350 (24.61)	10/249	8/101	2.00 (0.76–5.25)	0.160	0.090
Q2	350 (24.61)	10/246	14/104	3.37 (1.50–7.58)	**0.003**	
Q3	359 (25.25)	16/260	9/99	1.43 (0.63–3.23)	0.395	
Q4	363 (25.53)	22/268	6/95	0.81 (0.33–2.00)	0.647	

HR, hazard ratio; CI, confidence interval. Bold: Statistically different.

Among the metabolic profile subgroups, uric acid quartile analysis showed a significant interaction (*P*-interaction = 0.023): patients in the first quartile (lowest uric acid level) had a 6.5-fold higher risk of fatty liver disease (HR = 6.50, 95% CI: 2.75–15.34, *P* < 0.001). Diabetes stratification showed a stronger fatty liver association in non-diabetic patients (HR = 1.94, *P* = 0.015; diabetic patients HR = 0.90, *P* = 0.764), with a critically significant interaction (*P*-interaction = 0.073). In the lipid subgroups, the risk was significantly increased in the LDL ideal level group (HR = 2.19, *P* = 0.015) vs. the normal TC group (HR = 1.85, *P* = 0.017).

Liver function-related indicators showed differential associations: patients in the first tertile of ALT (lowest level) had a 4.38-fold higher risk of fatty liver (*P* = 0.047), while the third tertile of AST had the highest risk (HR = 3.80, *P* = 0.006). Bilirubin analysis showed a significant increase in risk in the first two tertiles of direct bilirubin (HR = 2.16, *P* = 0.047; HR = 2.34, *P* = 0.037), but total bilirubin was significant only in the second tertile (HR = 3.37, *P* = 0.003).

## Discussion

In the 5-year retrospective cohort study of railway safety staff, we identified that MAFLD was significantly associated with an increased risk of incidence of gallstones compared to the control group (HR = 1.71, 95% CI: 1.13–2.58). To our knowledge, this is the first study to verify the association between MAFLD and incidence of gallstones using longitudinal follow-up data in a population with special occupation of railway safety staff. Our findings extend beyond previous cross-sectional evidence and provide new insights into the relationship between MAFLD and gallstones, with implications for targeted prevention strategies.

The observed association between MAFLD and incidence of gallstones in railway safety staff cohort provides further evidence supporting the metabolic interplay between hepatic steatosis and biliary disease. While previous studies have predominantly focused on general populations or clinical cohorts, our findings highlight unique risk patterns in an occupational group characterized by high-stress environments and irregular lifestyles (23). In a propensity-matched analysis of 7,922 adults from the 2017–2020 National Health and Nutrition Examination Survey (NHANES), the presence of MAFLD increased the odds of GD by 49% (adjusted OR = 1.49; 95 % CI: 1.27–1.75) after adjustment for BMI (24). Similarly, a prospective cohort study of 58,862 participants in China revealed that metabolically healthy obese people had a 1.95 times higher risk of developing gallstones than metabolically healthy normal-weight people (25).

Previous experimental studies previously have shown that a high-fat diet induces hepatic lipid accumulation and alters bile composition. Six weeks of a high-fat diet increased total cholesterol and triglycerides by 211 and 231%, respectively, and significantly activated sterol regulatory element-binding proteins (SREBPs), suggesting activation of the lipid synthesis pathway and an increase in bile cholesterol, which is directly related to gallstone formation (26) -fat (LD) diet increased biliary cholesterol secretion two-fold in mice and significantly elevated the cholesterol saturation index (CSI), which directly led to increased gallbladder cholesterol stone formation (27) findings suggest that dysregulated lipid metabolism in fatty liver creates a biliary environment that favors gallstone formation. Lipid disorders commonly associated with insulin resistance in patients with fatty liver may further promote gallstone formation by altering bile acid metabolism (28, 29). In addition, elevated leptin levels in patients with fatty liver disease may impair gallbladder contractility, leading to cholestasis and stone formation (30).

The risk of gallstones in the participants of the present study was predominantly male, which contrasts with epidemiologic data showing female susceptibility to gallstones, and may reflect the impact of gender differences in occupational stress on metabolic dysregulation in railway safety staff (31). We hypothesize that this may reflect the negative impact of occupational factors (such as shift work, circadian rhythm disruption, and high stress) on metabolic homeostasis, thereby amplifying the role of MAFLD in the risk of gallstones among male railway safety staff. The smaller sample size of women in this study may also be one possible reason for the discrepancy with other research findings. However, this interpretation requires validation based on additional occupational exposure data and prospective studies. A particularly novel finding is that MAFLD patients in the lowest uric acid quartile have a dramatically elevated 6.5-fold risk, suggesting that hypouricemia may paradoxically promote gallstone formation in the setting of hepatic steatosis. This view is consistent with emerging evidence that uric acid, in addition to its role in gout, regulates oxidative stress and lipid peroxidation-a key process in cholesterol crystallization (21). Mechanistically, low uric acid levels may impair antioxidant defenses, allowing reactive oxygen species to promote cholesterol nucleation in bile, but this hypothesis needs to be tested in experimental models (32). Furthermore, stratified analyses revealed differential effects of MAFLD on gallstone risk across varying levels of TC, LDL-C, HDL-C, and triglycerides, underscoring the central role of lipoprotein metabolism in gallstone pathogenesis. In particular, the overproduction of cholesterol-rich LDL by the steatotic liver may directly raise biliary cholesterol saturation, thereby accelerating gallstone formation (33).

In our study, there was no significant association between BMI and gallstones, although there was also a strong association in the general population, which may reflect occupational confounders such as high levels of physical activity counteracting the effects of obesity (34, 35). In the setting of MAFLD, mild to moderate hepatic injury can indicate GDrisk by elevated ALT and AST. A meta-analysis showed that the OR of gallstones increased by 7% for every 10 U/L increase in ALT, and the pooled OR of elevated AST was 1.19. The mechanism is as follows: increased permeability of hepatocyte membrane to ALT/AST leakage, accompanied by bile acid synthesis obstruction and bile cholesterol supersaturation; ALT/AST is also positively correlated with insulin resistance and inflammation, and can amplify the nucleation environment. Therefore, ALT/AST is not only a marker of liver injury, but also a simple predictor of GDsusceptibility in patients with fatty liver (36) association of direct bilirubin (DBil) and indirect bilirubin (IBil) with MAFLD and the development of gallstones may be due to the fact that these two markers are not only signaling for cholestasis and hepatocellular damage, but may also influence cholesterol crystallization through two pathways: oxidative-antioxidant homeostasis and lipid metabolism (37, 38).

Our research has several limitations. Firstly, as the study population was occupationally exposed, the generalizability of the findings is somewhat limited. Future studies should examine these associations among different occupational groups to assess the broader applicability of our research results. Secondly, the retrospective cohort of this study was established based on physical examination data for up to 5 years. Although the completeness and accuracy of the data was relatively high, confounding factors such as smoking and alcohol intake can affect the veracity of responses due to the ban on alcohol and tobacco by railroad safety staff to the extent that they were not collected through questionnaires or interviews. Although the study population was highly homogeneous, these potential confounding factors should be further considered in clinical interpretation and future research. Finally, due to baseline inclusion reasons, the baseline GD population was insufficient in our research design, making it impossible to determine the causal relationship between gallstones and the incidence of MAFLD. The bidirectional association between the two was not confirmed. Early inclusion in cohort studies for observation and more prospective studies on different populations are needed to confirm the causal relationship between MAFLD and incidence of gallstones relationships.

In conclusion, our study advances the understanding of MAFLD and incidence of gallstones relationships by identifying occupational and metabolic modifiers of risk. In this longitudinal cohort analysis, lipid, uric acid, and bilirubin levels all affected the risk of gallstone development with MAFLD. These findings support the consideration of integrative interventions with other metabolic markers within the existing MAFLD management framework. Follow-up research directions should focus on elucidating mechanistic studies of the pathophysiologic determinants of MAFLD combined with multiple metabolic factors affecting gallstone occurrence, and expanding the observed population to more specifically exposed populations of interest.

## Data Availability

The raw data supporting the conclusions of this article will be made available by the authors, without undue reservation.
